# Hybrid deep learning and feature optimization approach for early detection of multiple sclerosis

**DOI:** 10.3389/fnhum.2025.1685580

**Published:** 2026-01-12

**Authors:** Nandini Anam, Sharief Basha S., Chiranji Lal Chowdhary

**Affiliations:** 1 Department of Mathematics, School of Advanced Sciences, Vellore Institute of Technology, Vellore, Tamil Nadu, India; 2 Department of Analytics, School of Computer Science and Engineering, Vellore Institute of Technology, Vellore, Tamil Nadu, India; 3 Department of Software and Systems Engineering, School of Computer Science Engineering and Information System, Vellore Institute of Technology, Vellore, Tamil Nadu, India

**Keywords:** multiple sclerosis, MRI, feature extraction, CNN, whale optimization algorithm (WOA)

## Abstract

The healthcare field increasingly relies on autonomous systems for the detection and analysis of Multiple Sclerosis (MS) to minimize diagnostic delays, resource burdens, reduce the progression of disability, and enhance clinical decision-making efficiency. Such systems ensure accurate and timely treatment, ultimately improved patient outcomes. In this study, a hybrid framework combining deep learning-based feature extraction, metaheuristic feature selection, and machine learning (ML) classifiers is proposed for accurate MS classification. All MRI images were preprocessed using Contrast Limited Adaptive Histogram Equalization (CLAHE), resizing, and normalization to enhance contrast and standardize the input dimensions. Deep features were extracted using the pretrained VGG16 convolutional neural network (CNN), in which the fully connected layers were removed, and the convolutional base was used to obtain high-dimensional features per image. To reduce dimensionality and improve classification performance, the Whale Optimization Algorithm (WOA) was employed to select the most discriminative subset of features using a Support Vector Machine (SVM)-based fitness function. Multiple classifiers were then trained and evaluated using the optimized feature set. Among them, the Artificial Neural Network integrated with WOA (ANN+WOA) achieved the highest classification accuracy of 98%, demonstrating the potential of the proposed model for reliable, efficient, and automated MS diagnosis.

## 1 Introduction

**FIGURE 1:**
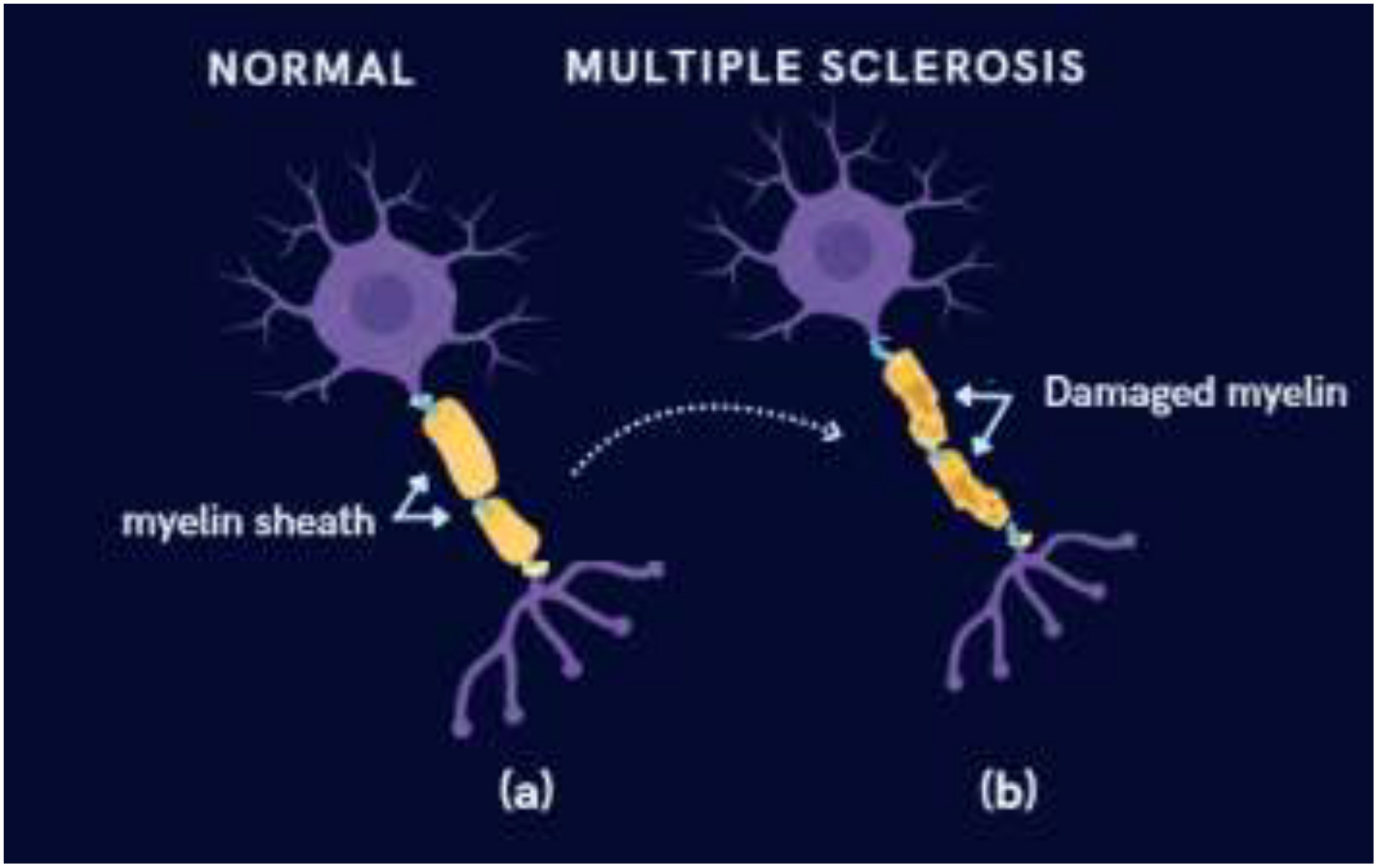
Difference between (a) healthy (intact) myelin and (b) damaged myelin associated with multiple sclerosis.

Multiple sclerosis is a long-term autoimmune disorder that damages the myelin sheath covering nerve fibers as seen in Figure 1, leading to the formation of lesions in the brain’s white matter (Steinman, 1996). Moreover, as an autoimmune disorder, MS leads to inflammation and harm to the myelin sheath, a protective layer around nerves, which impedes effective communication between the brain and the rest of the body. Approximately 3.59 out of 100,000 people worldwide suffer from MS (Szekely-Kohn et al., 2025). It is most commonly diagnosed in young adults between the ages of 20 and 45 and is more prevalent in women. The myelin sheath plays a crucial role in shielding nerve fibers and facilitating the rapid transmission of electrical signals between the brain and other parts of the body. This results in impaired signal transmission and progressive neurological deficits such as visual disturbances, motor impairment, and cognitive dysfunction. The disease typically begins as relapsing–remitting MS (RRMS) and may advance to secondary progressive MS (SPMS), marked by continuous disability accumulation. Early diagnosis and accurate classification of MS are clinically significant, as timely treatment can slow disease progression and improve quality of life. When demyelination occurs, it disrupts this signal conduction (Zhao and Jacob, 2023). The symptoms of multiple sclerosis differ among individuals but commonly include fatigue, muscle weakness, vision disturbances, depression, and balance issues. MRI, currently the most commonly used medical imaging method, plays a crucial role in detecting and diagnosing this condition (Sadeghibakhi et al., 2022).

Several potential risk factors have been identified as contributing to the development of MS, including a history of Epstein-Barr virus infection, geographic location based on latitude, the month of birth, and specific genetic markers (Barrie et al., 2024; Kurtzke et al., 1979). Similar to other autoimmune diseases, MS shows a clear gender disparity, affecting women approximately three times more often than men (Szekely-Kohn et al., 2025). Although the precise origin is still unknown, it is thought to be influenced by a number of variables, including viral infections, environmental factors, and genetics (Sadeghibakhi et al., 2022).

An MRI is one of the most important imaging modalities used in the medical field. especially MRI, have significantly enhanced our understanding of Multiple Sclerosis. High sensitivity is offered by MRI for the early identification of white matter inflammatory demyelinating lesions. There are several kinds of weighted MR images, such as sequences that are FLAIR-weighted (FLAIR-w), T1-weighted (T1-w), and T2-weighted (T2-w) (Zhao and Jacob, 2023). The three main anatomical planes in MRI and other medical imaging are: the body (or an organ) is divided horizontally by the axial plane, creating slices that divide the upper and lower components. Each axial picture in a brain MRI represents a “slice” seen from above, enabling measurements of lesion load, cortical thickness, and ventricular size at progressively higher elevations. The left and right parts of the body are separated by the vertical sagittal plane (Gray et al., 2008; Moore and Dalley, 2018). While parasagittal slices display lateral white-matter tracts and cortical areas, a midsagittal slice (across the midline) displays midline structures like the corpus callosum and brainstem. The anterior (front) and posterior (back) perspectives are separated by the coronal plane, which likewise extends vertically. The hippocampus, posterior fossa anatomy, and frontal and temporal lobes may all be shown in one continuous view with coronal MRI slices (Bushberg and Boone, 2011).

To assess the dynamics and course of the illness, it is crucial to accurately identify and segment MS lesions in MR imaging (Rolak, 2003). Artificial intelligence, particularly machine learning, has seen widespread adoption across various domains, including the field of medicine. Over the past decade, the application of ML in the study of neurological disorders has grown significantly Multiple machine learning techniques have been used to classify MS (AlSaeed and Omar, 2022). Recent developments in ML, DL, and image processing approaches seem to hold promise for automating the illness diagnosis procedure to enhance MS disease detection (Vázquez-Marrufo et al., 2023). Identification of neurological disorders is now feasible because to recent advancements in artificial intelligence (AI), particularly in ML, DL, and computer vision (Vijayan and Chowdhary, 2025).

**FIGURE 2:**
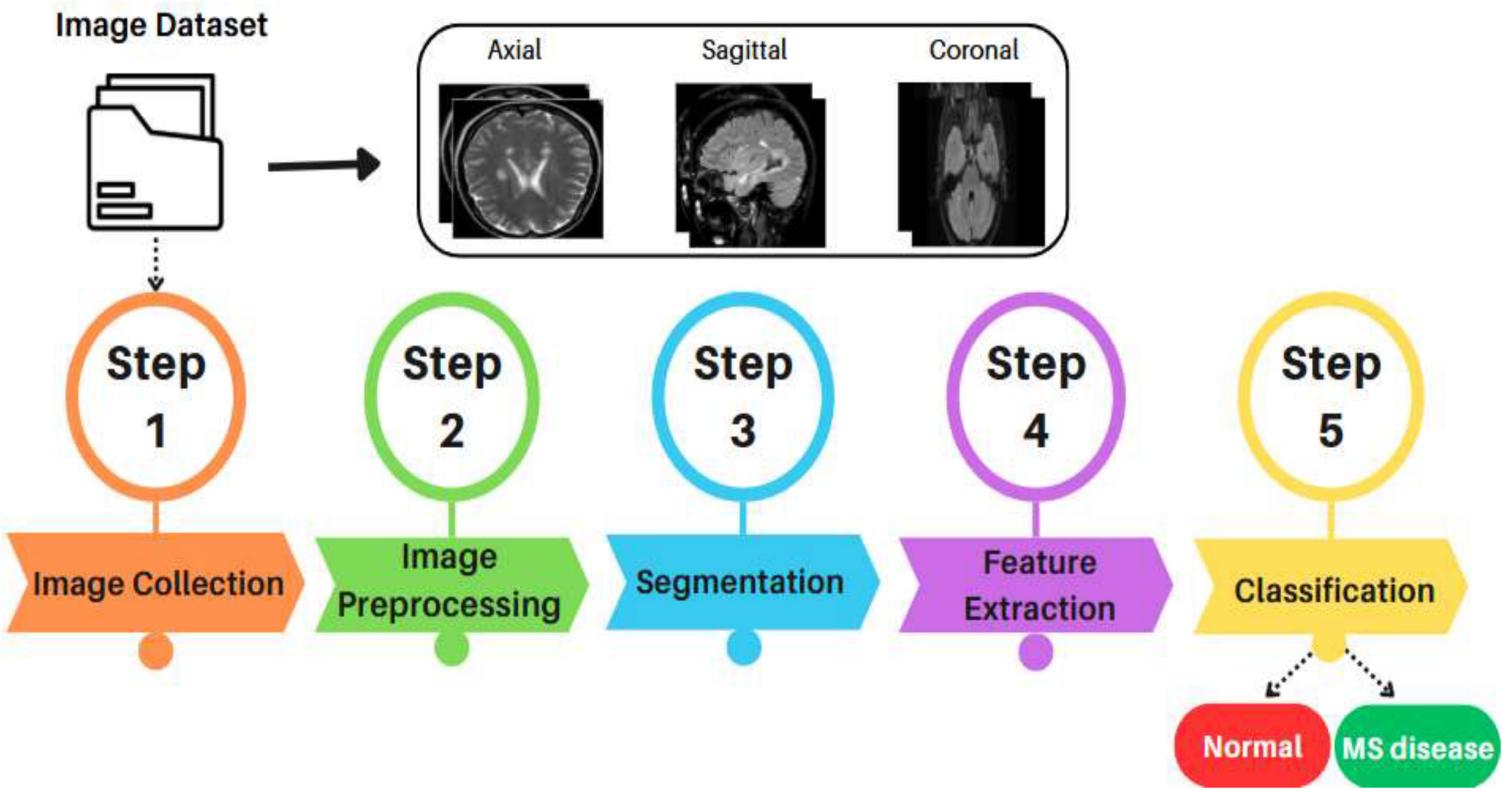
Basic procedure for classifying images.

The fundamental steps of picture categorization utilizing the MS illness dataset are shown in Figure 2. MRI images can be gathered directly from clinical sources or from pre-existing medical imaging libraries as part of the first phase. The next step is picture pre-processing. To increase picture quality and lessen distortion, common pre-processing methods including contrast enhancement and normalization are used. Resizing, grayscale conversion, noise reduction, normalization, binarization, and contrast enhancement are common procedures at this stage that get the pictures ready for additional examination and categorization (Dhanka and Maini, 2025).

Employing VGG16 as the sole feature extractor provides a simple yet powerful means to capture multiscale lesion patterns in MS MRI scans. This approach removes the need for manual feature engineering while enhancing both classification efficiency and accuracy, effectively distinguishing MS-related abnormalities from healthy tissue (Hu et al., 2025).

Authors in Daqqaq et al. (2024) used 2D–3D convolutional neural networks on MRI data to detect, categorize, and segment MS lesions. By combining performance measures from several investigations, including accuracy, sensitivity, specificity, and Dice Similarity Coefficient (DSC), they discovered that Convolutional Neural Network-based MRI demonstrated a strong DSC segmentation and very high lesion detection rates and classification accuracy with 98.81%. Placidi et al. (2025) describe an automated system that mimics expert radiologists by explicitly modeling diagnostic uncertainty, using several CNNs, each trained to identify lesions with knowledge of their spatial continuity and anatomical context, and combining their results using an ensemble classifier.

This approach integrates a bio-inspired optimization technique with deep learning to enhance diagnostic performance. Specifically, we employ a CNN_WOA framework, which combines CNNs for deep feature extraction with the WOA for optimal feature selection. This hybrid methodology is designed to improve diagnostic accuracy while reducing computational complexity, providing a robust and automated solution for the early detection and classification of MS.

## 2 Motivation and contributions

### 2.1 Motivation

In medical image analysis, especially for conditions like MS, it is very important to select the most relevant features from large and complex data. This helps improve the accuracy of disease detection and reduces the time and resources needed. Traditional methods often struggle to deal with the complex and non-linear nature of MRI images. The WOA, which is inspired by the hunting behavior of humpback whales, offers an effective way to select the best features. WOA works well because it can explore different possibilities while also focusing on the best solutions. When WOA is combined with features extracted from deep learning models like CNN, it helps boost the performance of classifiers such as SVM, ANN, and Random Forest. This leads to more accurate and faster diagnosis of MS using MRI scans.

### 2.2 Contributions

- First, we provide Conducted experiments on MS MRI datasets with separate training and testing folders, making the approach robust and clinically applicable.
- Second, we Proposed a novel hybrid framework that integrates CNN feature extraction with WOA metaheuristic algorithm for effective feature selection in MRI-based MS classification. Applied WOA to identify the most informative features from high-dimensional CNN outputs, significantly reducing feature space while improving classification accuracy.
- Thirdly, Performed a comprehensive comparison of several machine learning classifiers using selected features, demonstrating improved performance across models.

## 3 Related work

This section highlights key research and discoveries, emphasizing that timely detection plays a crucial role in delaying the advancement of MS. MRI plays a crucial role in identifying MS-related brain abnormalities, while also demonstrating the advancements in computationally intelligent systems for medical diagnosis. Researchers have automatically extracted discriminative characteristics from MRI images using deep learning models, namely CNNs (Krizhevsky et al., 2017; Litjens et al., 2017).

Valverde et al. (2017) enhanced sensitivity by creating a cascaded CNN system for MS lesion detection and segmentation. Aslani et al. (2019) suggested a multi-branch CNN architecture specifically designed for MS lesion segmentation, which demonstrated strong performance in a variety of MRI modalities. Nevertheless, high-dimensional feature vectors produced by CNN models frequently have the potential to impair the effectiveness and precision of conventional classifiers.

Studies have shown that the quality of datasets plays a major role in the results of empirical research, especially in software engineering (Mockus, 2008). Many researchers rely on publicly available datasets for their studies because they are easy to access. However, even though datasets are crucial for research, only a few studies consider their quality (Li et al., 2019; Rosli et al., 2016). If the datasets have quality issues, they can lead to misleading or classification unreliable conclusions. Especially MRI dataset aids in the detection, analysis, and of disorders in medical research and diagnosis.

In this research, we present a unique hybrid technique to successfully detect MS using brain MRI images by combining CNN-based deep feature extraction with WOA-based optimum feature selection. Machine learning methods including SVM, ANN, Random Forest, and KNN are then used for classification.

### 3.1 Image processing and techniques

The rapid growth of machine learning and artificial intelligence has enabled the use of image processing and classification techniques for the automated detection of neurological disorders. In particular, these technologies can be applied to analyze brain MRI scans and support the early and accurate diagnosis of MS, reducing the reliance on manual interpretation and improving clinical decision-making. DL, which is a type of ML, uses layers of neural networks to automatically learn the important features needed to detect diseases more accurately (Rana and Bhushan, 2023). CNNs are one class of deep learning models that have demonstrated superior performance in image-based tasks such as classification, object recognition, and segmentation.

CNNs have been widely applied in real-life tasks such as analyzing videos, understanding language, recognizing patterns, and processing speech. In CNNs, hierarchical connections with automated self-training and weight sharing are the most important aspects. The functions of semi-connected and fully connected layers differ, and they offer a suitable setting for both feed-forward and backward error propagation during the training process (Koushik, 2016; Liu, 2018).

Authors in Fiorini et al. (2015), A ML model was designed to evaluate clinical information and monitor the progression of Multiple Sclerosis. The primary objective was to differentiate between the benign and progressive types of the condition. Several classification algorithms were applied, such as Regularized Least Squares (RLS), Logistic Regression (LR), K-Nearest Neighbors (KNN), Ordinary Least Squares (OLS), and Linear Support Vector Machine (SVM). Researchers have also explored the combination of the fusion of CNNs and conventional classifiers like SVMs. CNNs have demonstrated strong performance in disease detection from images, thanks to their capacity to autonomously learn spatial features. The analysis utilized a dataset comprising 457 patient records, encompassing a total of 91 distinct features.

Authors in Zhang et al. (2021), A CNN model was constructed using MRI scans to categorize individuals into three classes: Relapsing-Remitting Multiple Sclerosis (RRMS), Secondary Progressive Multiple Sclerosis (SPMS), and healthy controls. The dataset comprised FLAIR, T1-weighted, and T2-weighted images, total 135 scans for each modality, obtained from 19 MS patients and 19 individuals without the disease.

### 3.2 Feature extraction techniques

Extracting features plays a crucial role in the analysis of medical images, particularly in disease diagnosis using MRI scans. In image processing, feature extraction refers to the identification and quantification of significant patterns or attributes within an image that can aid in classification and interpretation. Traditional methods focus on features related to texture, shape, intensity, and color to represent essential image characteristics. MRI illness diagnosis frequently uses first-order statistics, which characterize the intensity distribution within a ROI (Region of Interest). Handling such high-dimensional features can be computationally demanding, especially when employing deep learning architectures like ResNet.

Measures the average brightness in the regions shown below as Equations 1, 2.


$$\mathrm{Mean}\,\mathrm{Intensity}\,\left(\mu \right)=\frac{1}{N}\,\sum_{i=1}^{N}x_{i}$$ (1)

$$\mathrm{Standard}\,\mathrm{Deviation}\,\left(\sigma \right)=\sqrt{\frac{1}{N}\,\sum_{i=1}^{N}{\left(x_{i}-\mu \right)}^{2}}$$ (2)

Standard deviation is used as a feature because it indicates intensity variability, which helps differentiate between healthy and diseased brain tissues in MRI scans.

The spatial distribution of pixel intensities within an image region is described by texture characteristics, which aid in capturing patterns like regularity, roughness, granularity, and smoothness. These patterns are essential in brain MRI because they can identify lesions that differ in structural texture but not in intensity alone. We suggest a set of texture features that can be extracted from each grayscale spatial dependency matrix, as defined by the following mathematical expressions (Haralick et al., 2007; Kurniati et al., 2024).

Equation 3, referred to as contrast or inertia, quantifies the extent of intensity variation between neighboring image pixel elements. An elevated contrast measure reflects substantial differences in pixel intensities, whereas a low value suggests more uniform intensity. The contrast can be computed using the following expression (Razali et al., 2023).


$$\text{Contrast}=\sum_{i,j}{\left(i-j\right)}^{2}\,P\,\left(i,j\right)$$ (3)

In Equation 4, the energy metric rises when the matrix components exhibit uniformity or resemble each other. The formula for calculating energy is provided below (Kurniati et al., 2024; Park and Guldmann, 2020).


$$\text{Energy}=\sum_{i}\sum_{j}P\,{\left(i,j\right)}^{2}$$ (4)

Equation 5 represents entropy, which quantifies the amount of information or the degree of texture complexity present in an image (Guo et al., 2022; Kurniati et al., 2024).


$$\text{Entropy}=-\sum_{i}\sum_{j}P\,\left(i,j\right)\,\log\,P\,\left(i,j\right)$$ (5)

Closeness of the distribution to the diagonal represent in Equation 6:


$$\text{Homogeneity}=\sum_{i,j}\frac{p\;(i,j)}{1+|i-j|}$$ (6)

How correlated a pixel is to its neighbor as shown below Equation 7:


$$\text{Correlation}=\sum_{i,j}\frac{\left(i-\mu_{i}\right)\left(j-\mu_{j}\right)P\left(i,j\right)}{\sigma_{i}\,\sigma_{j}}$$ (7)

Where *i* and *j* represent the indices of the co-occurrence matrix, *P*(*i*, *j*) denotes the value at position (*i*, *j*)th within the matrix, μ_*i*_ and μ_*j*_ are the mean values of the row and column distributions, respectively, σ_*i*_ and σ_*j*_ correspond to the standard deviations of those row and column distributions (Kurniati et al., 2024).

Analyzing the size, form, and spatial properties of lesions found in brain MRI images requires the use of morphological data. These characteristics, which are often employed in both human and automated disease detection methods, aid in distinguishing diseased tissues from healthy tissue (Gonzalez, 2009).

With advancements in deep learning, CNNs have become widely used, as they can automatically learn complex and layered features from images without manual intervention. Overall, feature extraction transforms high-dimensional image data into compact and meaningful representations, making it easier for machine learning algorithms to learn effectively and produce accurate results. Because of their ability to identify complex visual patterns, these models are highly effective in extracting features that highly effective in extracting features that highly effective in extracting features that highly effective in extracting features that significantly improve classification accuracy. By leveraging pre-trained deep learning models to autonomously extract advanced features from images, the reliance on manual feature engineering decreases, while the accuracy of deep feature extraction using architectures like ResNet, InceptionNet, and VGG16 improves (Ahmad et al., 2021; Vijayan and Chowdhary, 2025).

### 3.3 Classification

Classification models are crucial parts of image-based analysis, especially when it comes to medical diagnostics like MRI scan illness detection. Image classification has shown significant potential in diagnosing various diseases, especially those affecting the brain, by leveraging advanced machine learning techniques to effectively analyze and interpret complex medical images (Singh et al., 2024).

Convolutional neural networks use the masked MRI slices to automatically train hierarchical, spatially invariant feature detectors (edges → textures → lesions). Equation 8 shows deep characteristics converted into a calibrated probability $\hat{y}$ for MS vs. control by the last sigmoid/softmax layer (Liu, 2018).


$$\hat{y}=\sigma \,\left(w^{T}\,z+b\right),\sigma \,\left(t\right)=\frac{1}{1+e^{-t}}$$ (8)

The final convolution + global-average-pooling feature vector is denoted by *z*.

*w*, *b* are the learned weights and bias of the final dense layer.

σ is applied, the logit becomes a likelihood that the scan is MS ($\hat{y}\approx 1$) or Control ($\hat{y}\approx 0$).

To discover non-linear correlations between CNN and texture characteristics, we input the CNN and texture information into a tiny neural network. Equation 9 represented as formula for new activation function.


$$h^{\left(l\right)}=f^{\left(l\right)}\,\left(W^{\left(l\right)}\,h^{\left(1-l\right)}+b^{\left(l\right)}\right)$$ (9)

*h*^(*l*)^: new activation.

*W*^(*l*)^: multiply the previous layer’s outputs by the weight matrix.

*f*^(*l*)^: non- linear function element wise.

*b*^(*l*)^: add the bias vector.

As defined in Equation 10, Support Vector Machines that use an RBF (Radial Basis Function) kernel to identify the maximum-margin boundary in a converted feature space perform very well in high-dimensional, small-sample scenarios (Vázquez-Marrufo et al., 2023).


$$\hat{y}=s\,i\,g\,n\,\left(\sum_{i=1}^{N}\alpha_{i}\,y_{i}\,e^{-\gamma \,{||x_{i}-x||}^{2}}+b\right)$$ (10)

α_*i*_ are the learned dual coefficients.

*y_i_* is the *label* of the ith training example and *b* being the bias.

k-Nearest Neighbors classifies a new MRI by finding the most similar training scans in feature space and letting them “vote” on the label, without fitting any global model. Therefore, when your fused MRI features are correctly normalized and highly discriminative, KNN is straightforward, clear, and efficient. Equation 11 formulates the normalized discriminative to closest training points.


$$\hat{y}=\arg\max_{c\in \left\{0,1\right\}}\sum_{x_{i}\,\varepsilon \,N_{k}\,\left(x\right)}1\left[y_{i}=c\right]$$ (11)

*N*_*k*_(*x*): is the set of the *k* closest training points to *x.*

1 [*y*_*i*_ = *c*]: counts how many neighbors belong to class *c.*

## 4 Proposed model

**FIGURE 3:**
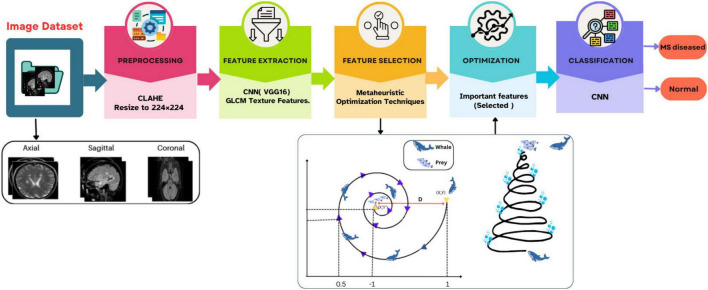
Workflow for proposed model.

The main aim of this study is to design a classification system supported by optimal feature selection to accurately identify MS using brain MRI images. MS is a neurological disorder that can severely affect motor and cognitive functions if not diagnosed in its early stages. Early and accurate detection is essential to slow disease progression, enhance treatment effectiveness, and improve patient outcomes. Traditionally, MS detection is done manually by experts analyzing MRI scans, which is time-consuming and may lead to inconsistencies. To address this, an automated approach is introduced to detect MS-related abnormalities in MRI images more efficiently. In this process, CNNs play a key role, as they are capable of learning detailed image patterns and performing precise classification when trained with sufficient and quality data. Algorithm 1 presents a detailed overview of the proposed model, while Figure 3 provides its visual representation.

Algorithm 1: A comprehensive description of the proposed model.

**Step 1:** Data Collection

 •Gather the brain MRI data to initiate the preprocessing stage.

**Step 2:** Image Preprocessing

 •Resize to 224 × 224 for CNN input.

 •Adaptive Histogram with Contrast Limitation (CLAHE) the purpose of equalization is to enhance the contrast of images.

**Step 3:** Feature Extraction

 •CNN (VGG16): Use pretrained model (without top layers), extract 25,088 features per image.

 •GLCM Texture Features.

**Step 4:** Feature Selection

 •WOA

 ∘Whale agents are initially positioned randomly across the search domain.

 ∘Compute the fitness value for every agent based on the objective function, often determined by how accurately it classifies data or its associated error.

 ∘The whale achieving the best fitness is selected as the reference point for the rest of the population.

**Step 5:** Classification

 •CNN learns important features from images on its own and uses them to carry out classification.

 •The trained CNN model is used to classify new images after they are processed and optimized through feature extraction.

### 4.1 Image dataset

**FIGURE 4:**
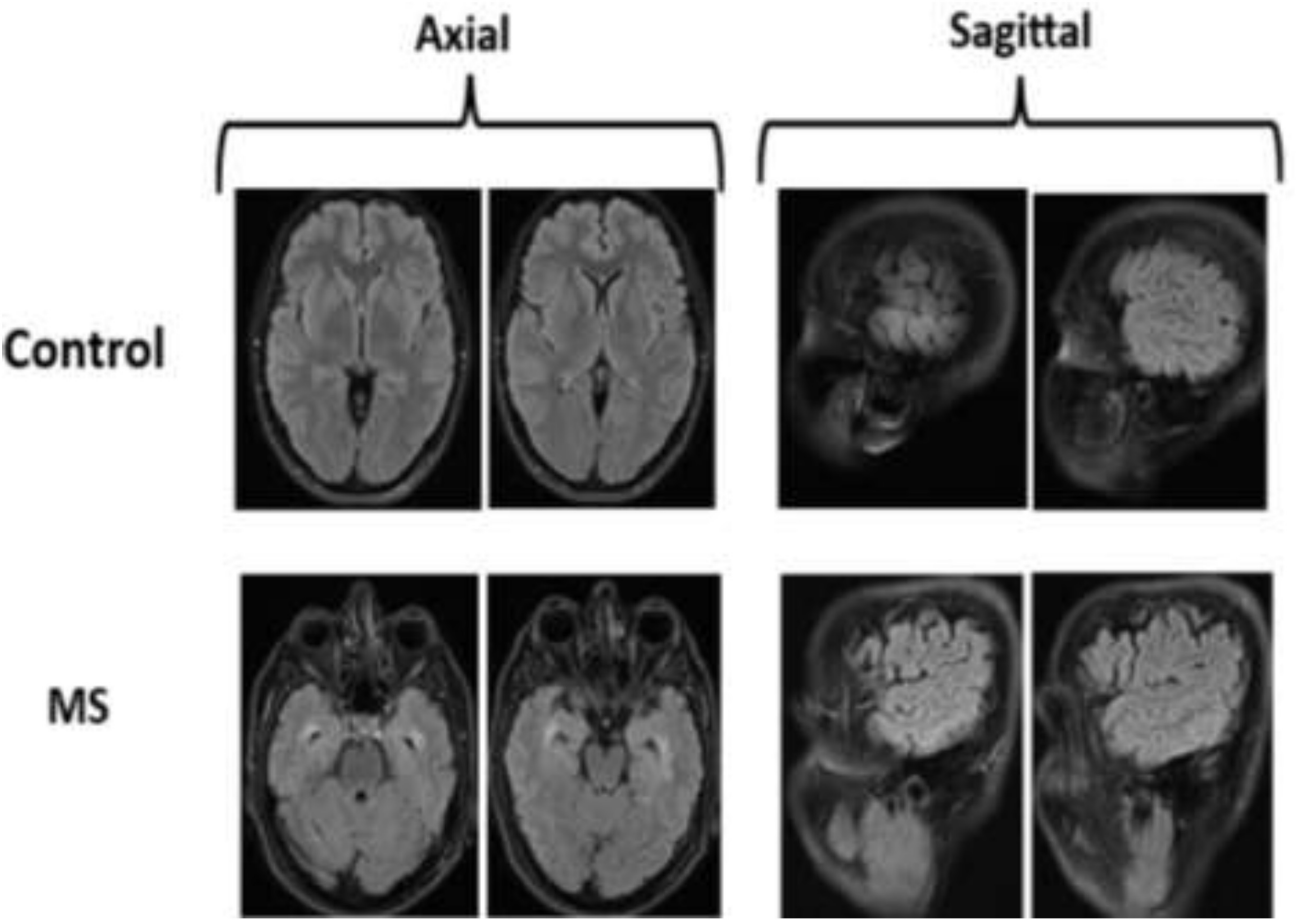
Sample images from archive.


The dataset used in this study contains a total of 3,427 MRI images, sourced from Kaggle. It is categorized into four classes as shown in Figure 4. To ensure consistency and improve the performance of the classification model, all images were normalized during preprocessing. The mathematical representation of the normalized image dataset is provided in Equation 12 below.

M=i{Ib⁢1,Ib⁢2,Ib⁢3,…Ib⁢i} (12)

Where

*M*_*i*_ input images related to MS present in the archive.

*I*_*b*_ represents the brain images included in the dataset.

*i* denotes the overall number of images stored in the archive.

### 4.2 Pre-processing

**FIGURE 5:**
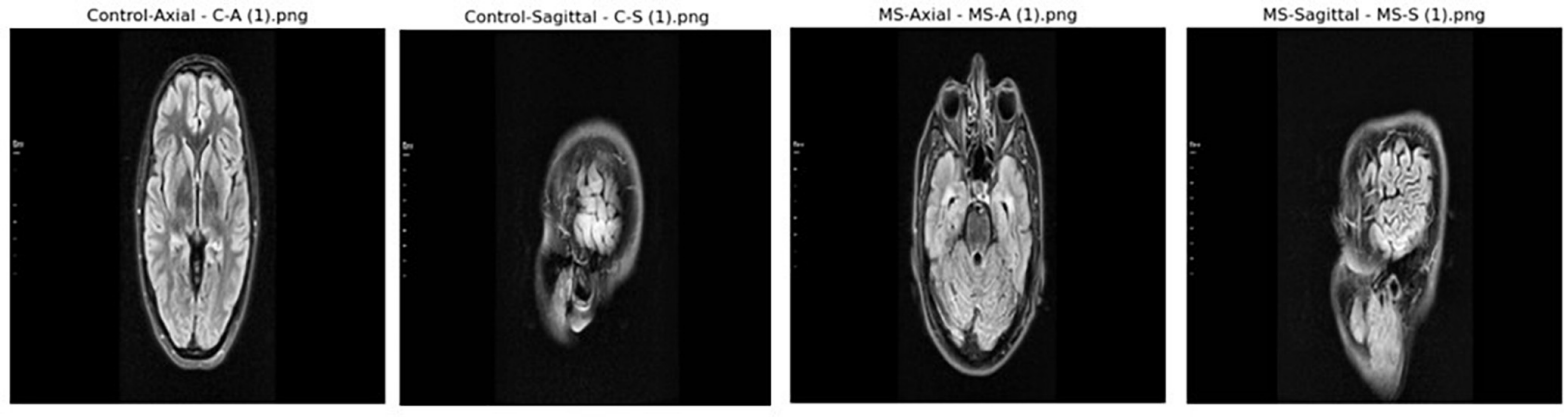
After applying CLAHE for input image.

To enhance the visibility and overall quality of MRI scans, a preprocessing stage is incorporated into the proposed model prior to feature extraction and classification. Initially, each image is processed using CLAHE, a method that improves local contrast and highlights critical structural features, particularly in areas with low intensity variations. CLAHE operates by adjusting pixel intensities within localized image regions, thereby enhancing important visual details. After contrast, GLCM techniques are used to extract texture-based features from the grayscale version of the improve images (Vijayan and Chowdhary, 2025). This preprocessing pipeline ensures that the model is trained on high-quality, feature-rich data, which is essential for accurate classification. Figure 5 presents the outcome after applying CLAHE to the input image.

Standardizing medical image dimensions through resizing is a fundamental step in preparing data for convolutional neural networks. This practice involves adjusting images to predetermined sizes, such as 224 × 224 pixels commonly used with the VGG16 architecture, to ensure uniformity across the dataset and compatibility with pretrained models. The inherent design of deep neural networks enables them to identify and extract multi-level spatial and contextual patterns, which means critical diagnostic features and lesion characteristics remain discernible despite dimensional adjustments.

Modern resizing techniques employ sophisticated interpolation methods like bilinear and bicubic algorithms to maintain image integrity during the transformation process. These approaches effectively minimize data loss that might otherwise occur during scaling operations. Research in neuroimaging has consistently demonstrated that standardizing brain MRI dimensions to conventional scales has negligible impact on either lesion detection capability or the predictive performance of classification systems when using deep learning for feature extraction (Hussain et al., 2025; Simonyan and Zisserman, 2014).

In mathematical form, CLAHE acts as a transformation over specific image tiles to enhance local contrast as Equations 13, 14 (Zuiderveld, 1994).


$$I_{C\,L\,A\,H\,E}\,\left(x,y\right)=C\,\left[I\,\left(x,y\right)\right]=I\,n\,t\,e\,r\,p\,o\,l\,a\,t\,i\,o\,n\left(T_{k}\,\left(I\,\left(x,y\right)\right)\right)$$ (13)

where


$$T_{k}\,\left(i\right)=\frac{\left(L-1\right)}{N_{k}}\,\sum_{j=0}^{i}\min\,\left(H_{k}\,\left(j\right),c\,l\,i\,p\,\_\,l\,i\,m\,i\,t\right)$$ (14)

*I*(*x*, *y*): original grayscale image

*I*′(*x*, *y*): enhanced image after applying CLAHE

∁:the CLAHE transformation operator

*T*_*k*_(*i*): transformation function in local

*L*: total number of gray levels

*H*_*k*_(*i*): histogram of gray levels in tile *k*

*N*_*k*_: number of pixels in the tile *k.*

### 4.3 Feature extraction

Feature extraction plays a vital role in image segmentation, as it helps identify key patterns associated with MS disease. This study uses VGG16, a popular CNN architecture as seen in figure that is well-known for its deep structure and robust performance in picture classification applications, for feature extraction. Here, VGG16 is used without the top (classification) layers after being pretrained on the ImageNet dataset. The convolutional and pooling layers of VGG16 process input MRI pictures, automatically learning and extracting significant characteristics including edges, textures, and high-level spatial patterns. The last convolutional block’s output is flattened into a feature vector that represents the input images deep learnt properties. These characteristics are then either fed into an optimization algorithm for feature selection or used for classification. With this method, the model may take use of high-dimensional, rich characteristics without the need for human involvement.

### 4.4 VGG16

**FIGURE 6:**
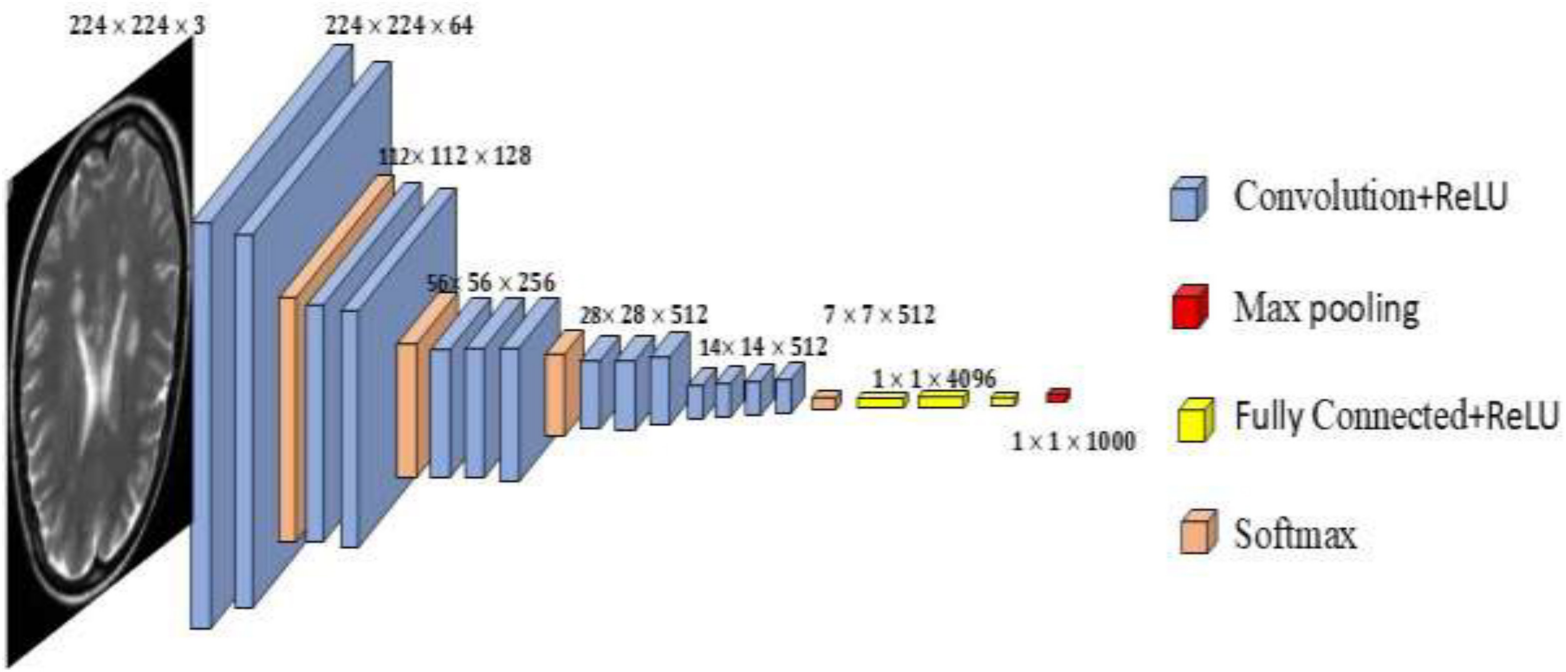
VGG16 architecture.

It represents a deep learning architecture based on convolutional neural networks, as shown in Figure 6, comprising 16 weight layers that include 13 convolutional layers and 3 fully connected layers. The network employs 3 × 3 convolutional kernels applied with a single-pixel stride, incorporating rectified linear unit (ReLU) activation functions to introduce nonlinear transformations. Five 2 × 2 max-pooling operations are interspersed between convolutional blocks, systematically reducing spatial dimensions while maintaining essential feature information. The architecture concludes with three densely connected layers, culminating in a softmax layer for classification purposes. In this implementation, the fully connected layers were excluded, and the output from the final convolutional block was flattened to generate a comprehensive feature vector for subsequent optimization and classification tasks (Hussain et al., 2025).

**FIGURE 7:**
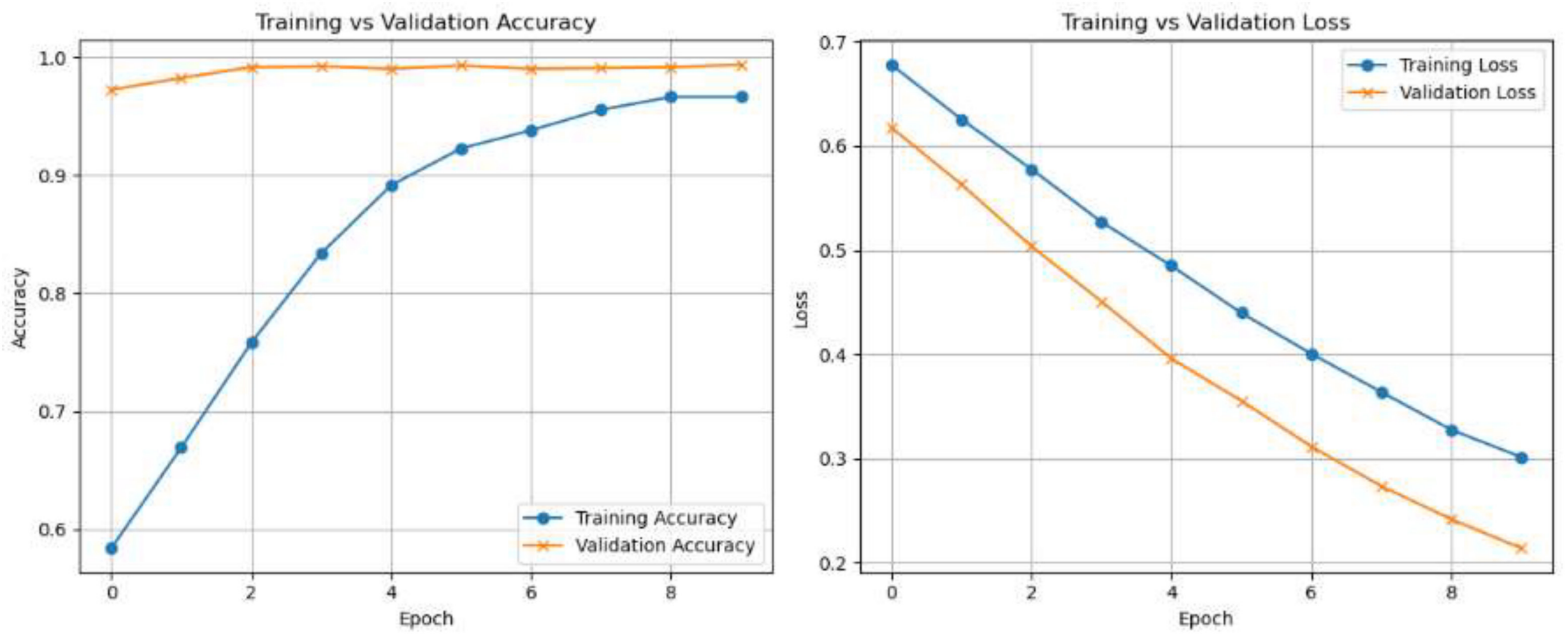
Training and validation loss trends for the CNN (VGG16) model. The steadily decreasing validation loss demonstrates good generalization and minimal overfitting.

The experimental findings show that the suggested CNN-based model, which makes use of the VGG16 architecture, performs very well in the classification of MRI images for the purpose of detecting multiple sclerosis. Strong generalization and efficient learning were demonstrated by the model’s peak validation accuracy of almost 99.29% across 10 training epochs and its constantly declining validation loss, which reached 0.1772 as seen in Figure 7 and the CNN (VGG 16) parameters and values are shown in Table 1.

**TABLE 1:** CNN (VGG 16) parameters and values.

Parameters	Values
Optimizer	Adam
Loss function	Binary crossentropy
Epochs	10
Batch size	32

In Table 1, Adam was picked because it works well and can change its learning rate on its own, which makes training go faster and be easy. Since binary crossentropy efficiently quantifies the discrepancy between projected probability and true labels, it is most appropriate for binary classification problems (e.g., disease vs. healthy). The complete training dataset is processed 10 times to optimize the weights of the model, which is known as 10 epochs. To balance memory utilization and computational performance during training, a batch size of 32 is employed.

The model’s stability and lack of overfitting are confirmed by the training and validation curves’ tight alignment. These results confirm the efficacy of utilizing CNNs for deep feature extraction in conjunction with appropriate preprocessing and class balancing to provide a dependable and precise diagnostic assistance system for MS classification.

### 4.5 Feature selection

Feature selection helps choose the most important features from a large set, making it easier and faster to identify diseases accurately. Metaheuristic optimization algorithms are a group of techniques that work by repeatedly evaluating objective functions, usually without the use of gradient information (Yang et al., 2013). In this study, the Whale Optimization Algorithm is used alone for feature selection to improve the identification of MS disease, leveraging its strength in exploring and selecting the most relevant features. WOA helps you find the “best subset” of features for disease classification, improving accuracy and speed, and is particularly effective for handling the large, complex feature sets produced by deep learning models.

#### 4.5.1 Whale optimization algorithm

**FIGURE 8:**
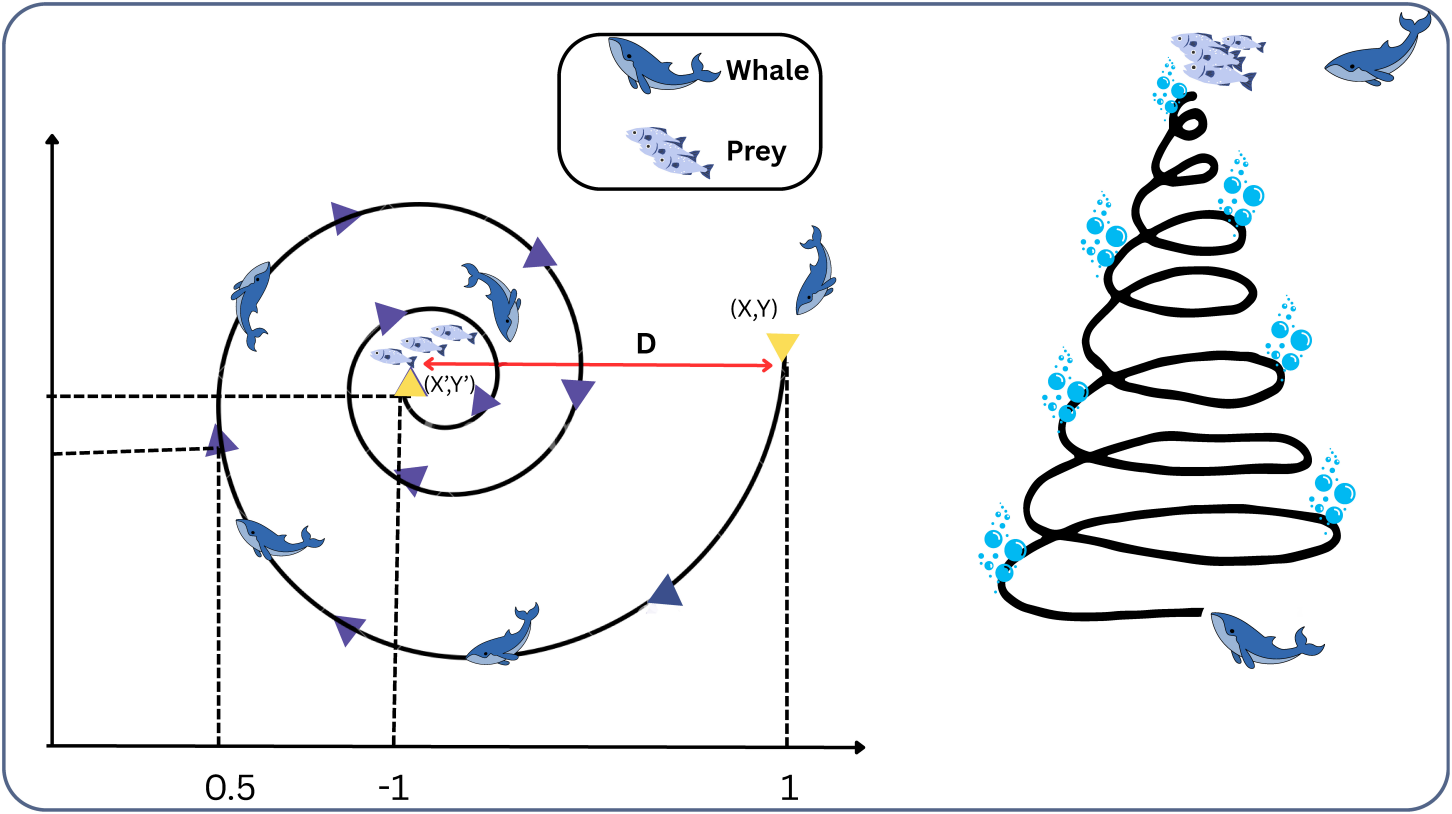
Whale optimization algorithm.

The WOA is a bio-inspired metaheuristic that replicates the distinctive foraging strategy of humpback whales, specifically their spiral bubble-net hunting method. This technique involves whales creating bubble rings to encircle and trap their prey. They typically target schools of small fish or krill near the water’s surface, creating spiral or “9”-shaped bubble patterns to trap and gather their prey. WOA operates through two core stages: exploration and exploitation. In the exploration stage, whales perform a global search to locate potential prey, whereas in the exploitation stage, they converge on the identified target using a spiral bubble-net motion, reflecting the natural hunting behavior of real whales. The algorithm alternates randomly between spiral motion and encircling techniques to update the positions of the search agents. Overall, WOA operates through three key stages: Locating the prey, surrounding it, and executing the attack, inspired by the natural hunting process of humpback whales (Mirjalili and Lewis, 2016). Figure 8 illustrates the behavior of the Whale Optimization Algorithm.

#### 4.5.2 Encircling prey

Once humpback whales identify their prey, they initiate a circular motion around it. Similarly, in WOA, the most optimal solution is assumed to be closest to the target, prompting other solutions to adjust their positions accordingly.


$$\vec{D}=|\vec{C}.{\vec{X}}_{b\,e\,s\,t}\left(t\right)-\vec{X}(t)|$$ (15)

Here Equation 15 represents the distance between current solution and best solution. Equation 16 represents the mathematical formulation of current iteration for best solution.


$$\vec{X}\left(t+1\right)={\vec{X}}_{b\,e\,s\,t}\,\left(t\right)-\vec{A}.\vec{D}$$ (16)

Where t indicates the current iteration, $\vec{A}$ and $\vec{C}$ are coefficient vectors, ${\vec{X}}_{b\,e\,s\,t}$ is the position vector of the best solution, according to Equations 17, 18.


$$\vec{A}=2\,\vec{a}\,{\vec{r}}_{1}-\vec{a}$$ (17)

$$\vec{C}=2\,{\vec{r}}_{2}$$ (18)

Where ${\vec{r}}_{1}$, ${\vec{r}}_{2}$ are random vectors in [0,1].

The balance is controlled by the parameter $\vec{a}$, which gradually drops from 2 to 0:

When $\left|\vec{A}\right|>1$ is the exploration and $\left|\vec{A}\right|<1$ is exploitation.

The parameter controlling convergence $\vec{a}$, It gradually declines in a linear fashion from 2 to 0 throughout the iterations, influencing the balance between exploration and exploitation phases. Here $a\,2-\frac{2\times t}{t_{m\,a\,x}}$, $\vec{r}$ is random vectors drawn from a uniform distribution within [0, 1] (Vijayan and Chowdhary, 2025).

#### 4.5.3 Bubble-net attacking method

Bubble-net feeding is a distinctive hunting behavior seen exclusively in humpback whales. In the WOA, this spiral movement is mathematically represented to simulate the optimization process. The following two approaches have been developed.

##### 4.5.3.1 Shrinking encircling mechanism

One of the key behaviors modeled in the WOA is the shrinking encircling mechanism, which imitates how humpback whales gradually move toward their prey. In the algorithm, this is achieved by linearly decreasing the convergence coefficient over the course of iterations. As a result, the search agents move progressively closer to the best solution found so far. Over time, they focus their search within a narrower region around this solution, allowing the algorithm to better refine its search and explore more promising areas in the solution space.

##### 4.5.3.2 Spiral updating position

The spiral updating position is one of the strategies used during the exploitation phase of the WOA. It replicates the spiral motion observed in humpback whales during their bubble-net feeding behavior to capture prey (Sharawi et al., 2017).

This movement is mathematically represented using an exponential spiral equation that combines both angular motion and distance to replicate the whale’s trajectory, as represented by Equation 19.


$$\vec{X}\,\left(t+1\right)=\vec{D}.e^{b\,l}.\cos\,\left(2\,\pi \,l\right)+{\vec{X}}_{b\,e\,s\,t}\,\left(t\right)$$ (19)

*l* is a random number in [–1,1].

*D* is $\vec{D}$ = $\left|\vec{C}.{\vec{X}}_{b\,e\,s\,t}(t)-\vec{X}(t)\right|$ represents the distance between the *i*th whale and the prey, which corresponds to the current best solution found.

#### 4.5.4 Search for pray

This stage mimics the natural hunting behavior displayed by humpback whales in their environment, where they are seeking across a large area and are uncertain of the precise location of their prey, by having whales (search agents) randomly navigate over the search space to locate promising regions rather than immediately targeting the best-known answer (Mirjalili and Lewis, 2016).

To simulate this mathematically, a random whale position ${\vec{X}}_{r\,a\,n\,d}$ is chosen, and the current whale’s position is updated using the Equation 20 that follows:


$$\vec{D}=|\vec{C}.{\vec{X}}_{r\,a\,n\,d}\left(t\right)-\vec{X}\left(t\right)|$$ (20)

$$\vec{X}\,\left(t+1\right)={\vec{X}}_{r\,a\,n\,d}\,\left(t\right)-\vec{A}.\vec{D}$$

Where

${\vec{X}}_{r\,a\,n\,d}\,\left(t\right)$ placement of a whale chosen at random.

$\vec{X}\,\left(t\right)$ the whale’s current location.

$\overset{↔}{X}\,\left(t\right)$ the vector of current location.

$\vec{A}$ an adjustment for step size.

*t* is the iteration index or current time.

### 4.6 Classification

CNNs are able to do classification directly through the use of fully linked layers and automatically extract hierarchical spatial information from pictures. As an alternative, deep features may be used to classify data using conventional machine learning models. With this hybrid technique, performance is frequently enhanced by combining the classification capability of ML algorithms with the feature learning strength of CNNs. Furthermore, information regarding subtle or hidden characteristics that could be present in a picture cannot be extracted using conventional manual procedures. On the other hand, DL-based image classification and identification techniques automatically extract picture characteristics using artificial neural networks (Shoaib et al., 2023).

The designed CNN architecture includes multiple layers tailored to capture and learn significant features from brain MRI scans. A resized image of 224 × 224 pixels is fed into the CNN and initially processed by convolutional layers (Liu et al., 2023). The feature maps are subsequently down sampled using max pooling layers, which reduce spatial dimensions while preserving the most important information. This helps to control overfitting and computational expense (Tayal et al., 2022). The final classification differentiating between MS and control cases is carried out by a fully linked layer after the retrieved features have been flattened.

The ANN, SVM, KNN, LR, and RF classifiers were used to assess the efficacy of the Whale Optimization Algorithm feature selection. Each model has unique learning properties and optimization tendencies. Here, ANN is a multilayer network that can capture complicated nonlinear interactions via iterative weight updates with backpropagation and nonlinear activation functions, allowing adaptive learning from the optimum feature subset. In contrast, the SVM creates an ideal hyperplane that optimizes the margin between classes and combined with WOA, the selection of discriminative features improves border separation and decreases classification error (Vázquez-Marrufo et al., 2023). The RF classifier is an ensemble of decision trees that perform feature bagging and random sampling to increase robustness and the addition of WOA refines the input feature space, resulting in better model stability and generalization.

Moreover, K-nearest neighbors approach categorizes data items by comparing them to neighboring samples in the feature space. The WOA improves this procedure by identifying and retaining just the most important distance measurement parameters. Such optimization reduces processing needs while simultaneously improving classification accuracy. Similarly, logistic regression (LR), which uses a sigmoid function to estimate class probabilities, benefits from WOA’s ability to reduce feature sets. This modification removes extraneous variables while enhancing model interpretability. By including WOA into both classification algorithms, the system achieves enhanced predicted accuracy, improved adaptability to novel data, and increased computing efficiency. Overall, the WOA integration guarantees that each classifier receives a concise and informative set of data, increasing accuracy and computing efficiency (Haralick et al., 2007).

**FIGURE 9:**
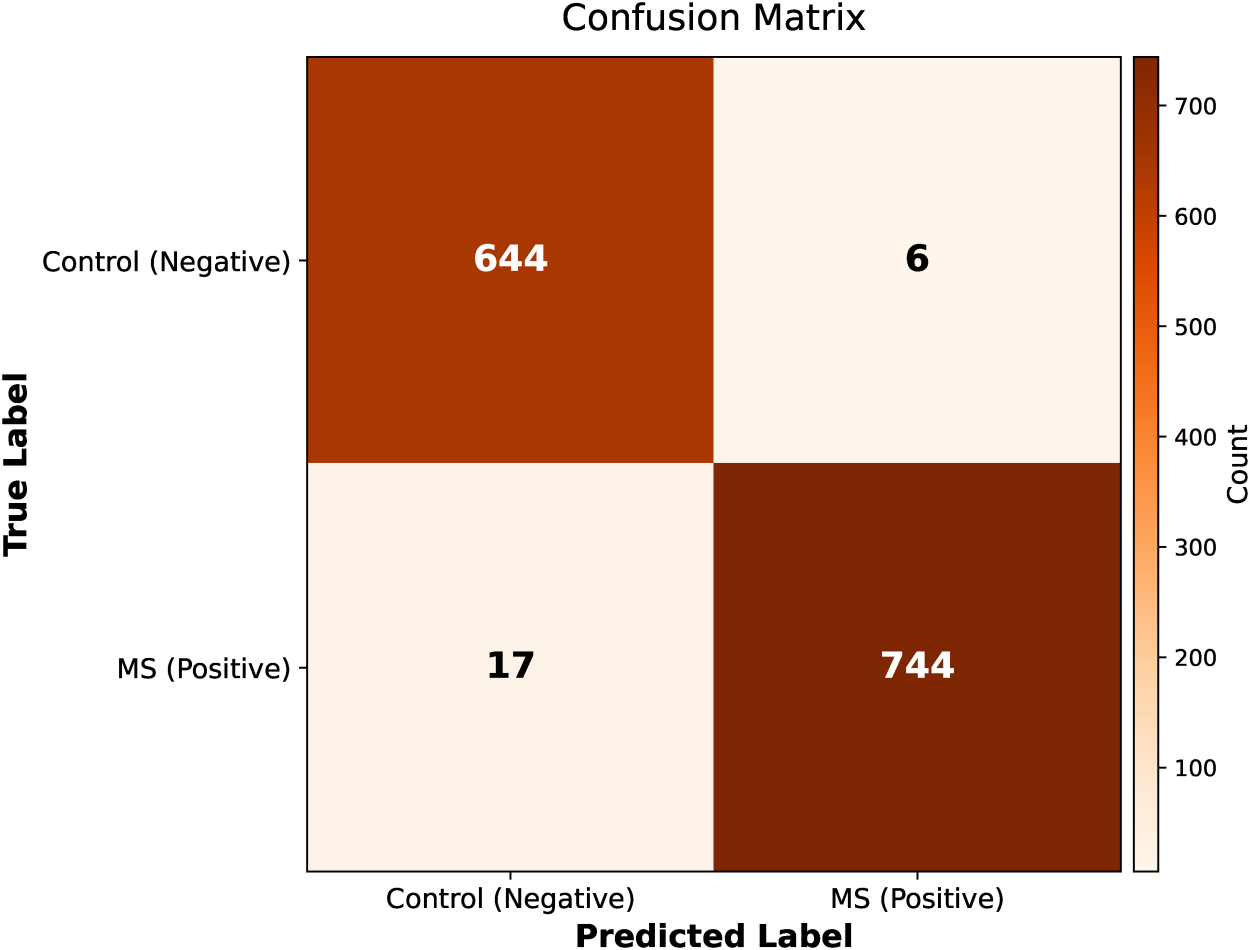
Confusion matrix depicting ANN classification performance.

Figure 9 the classification performance for Multiple Sclerosis and Control MRI images is displayed in the Confusion Matrix of the proposed Artificial Neural Network model. The model’s capacity to correctly identify MS patients was confirmed by its overall accuracy and good recall, and specificity.

## 5 Results and discussion

To evaluate the effectiveness of the proposed method, we used a dataset containing 3,427 brain MRI images related to Multiple Sclerosis, collected from Kaggle. The dataset is categorized into four classes. The pre-processing was applied to the images to ensure uniformity in size and contrast. Data was split into 80% training and 20% testing sets to evaluate model performance. The training set was used for feature extraction and model learning, while the test set was used to assess classification accuracy. CLAHE was first used to enhance image contrast, followed by feature extraction using the CNN (VGG16) model. The most relevant features were then selected using the Whale Optimization Algorithm. In this study, feature extraction was performed on preprocessed MRI images without segmentation. A pretrained VGG16 model was used to extract deep global characteristics that encompassed both healthy and diseased tissue attributes. The Whale Optimization Algorithm was used to optimize the deep feature vector generated by flattening the output of the last convolutional layer, retaining just the most discriminative features for classification.

Applying our deep learning framework, which integrates VGG16 and the Whale Optimization Algorithm, to entire MRI scans means it evaluates both significant (lesion) and non-significant (normal tissue) areas. Nonetheless, advanced CNNs like VGG16 are inherently structured to concentrate on the most critical parts of an image during training, a mechanism often referred to as spatial attention within the convolutional hierarchy. VGG16’s convolutional layers progressively learn to identify localized features such as edges, textures, and intensity changes, with deeper layers extracting more complex semantic details like tissue irregularities. Consequently, disease-related patterns become more pronounced, while less pertinent background information (such as standard gray and white matter) is naturally downplayed.

The method can be further refined by isolating the most critical characteristics that enhance classification through the application of the Whale Optimization Algorithm for feature selection. This approach proves effective even in the absence of initial segmentation, as WOA efficiently eliminates unnecessary or repetitive data points commonly found in healthy tissue areas. This strategy broadens the framework by integrating the strengths of deep neural networks with optimization methods, ultimately leading to more precise Multiple Sclerosis detection.

The ANN model, when integrated with WOA, outperformed the other classifiers in terms of overall performance. Table 2 provides a Performance comparison across multiple classification algorithms. The close alignment between training and validation accuracy confirms that the models generalize well to unseen data. The combined impact of deep CNN-based feature extraction, CLAHE preprocessing, and nature-inspired feature selection significantly contributed to the high classification performance.

**TABLE 2:** Performance comparison of classification techniques with feature selection.

Model	Accuracy (%)	Precision	Recall	F1-score
WOA+ANN	**99.71**	0.99	0.98	0.98
WOA+SVM	97	0.96	0.97	0.97
WOA+RF	94	0.95	0.93	0.93
WOA+KNN	97	0.95	0.98	0.97
WOA+LR	98	0.96	0.99	0.97

Bold value indicates the best performance among the compared methods for each evaluation metric.

The model’s effectiveness is assessed using evaluation metrics such as accuracy, sensitivity, and specificity, as defined in Equations 20–23, respectively (Lee et al., 2018; Reinke et al., 2021).


$$\text{Precision}=\frac{T\,P}{\left(T\,P+F\,P\right)}$$ (21)

$$\text{Recall}=\frac{T\,P}{\left(T\,P+F\,N\right)}$$ (22)

$$\text{Accuracy}=\frac{\left(T\,P+T\,N\right)}{\left(T\,P+T\,N+F\,P+F\,N\right)}$$ (23)

$$\mathrm{F1 score}=2*\frac{\left(\frac{T\,P}{T\,P+F\,P}\right)*\left(\frac{T\,P}{T\,P+F\,N}\right)}{\left(\frac{T\,P}{T\,P+F\,P}\right)+\left(\frac{T\,P}{T\,P+F\,N}\right)}$$ (24)

**FIGURE 10:**
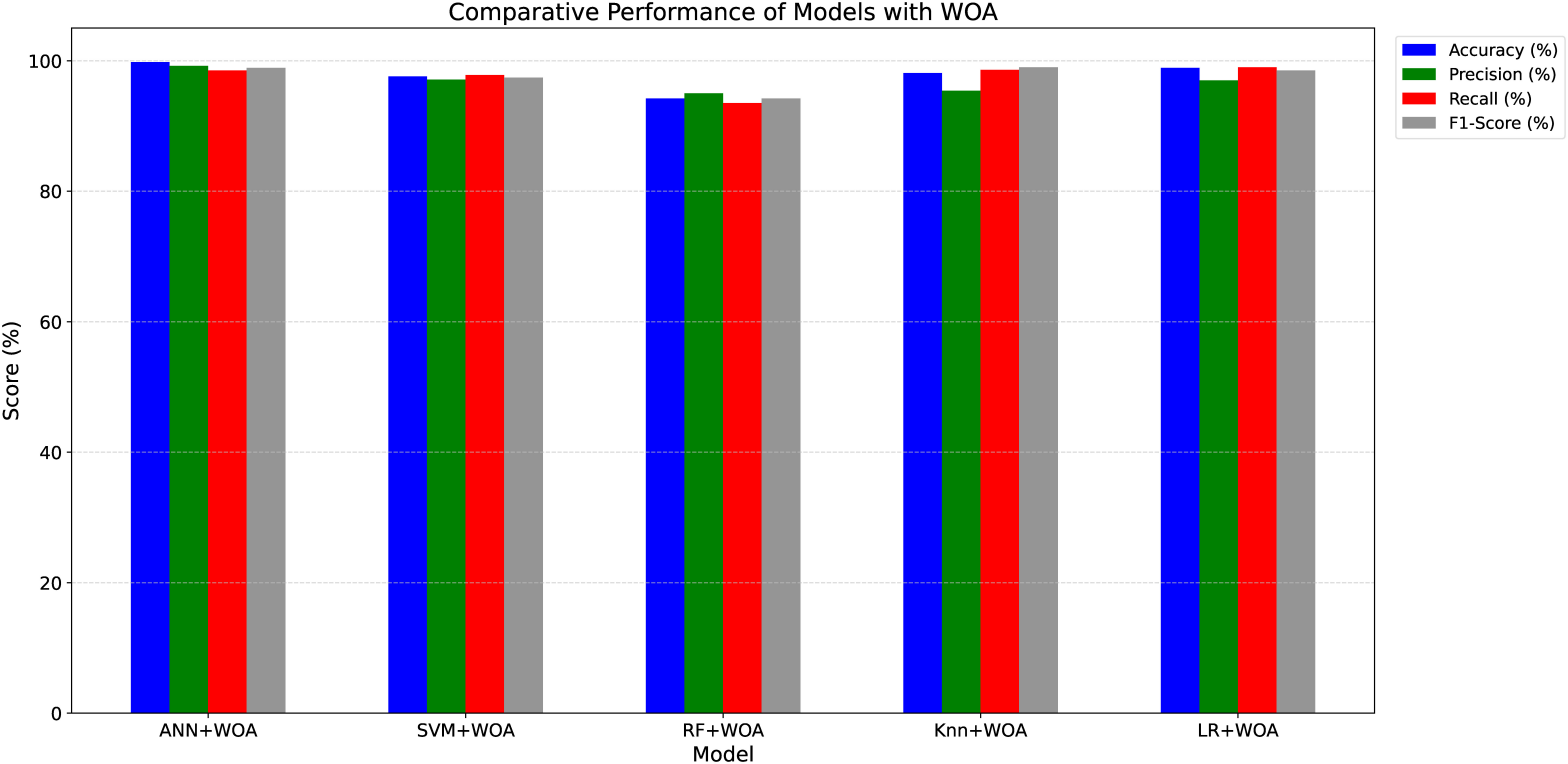
Model evaluation metrics.

Negative means were eliminated and positive means were determined in accordance with general guidelines. Because of this, a true positive (TP) indicates that a patient with MS has been accurately diagnosed, whereas a false positive (FP) indicates that healthy individuals were falsely recognized as healthy, true negative (TN) indicates that healthy individuals were accurately identified as such, while false negative (FN) indicates that MS patients were misidentified as healthy (Zhang et al., 2016). Figure 10 illustrates the comparison of performance metrics for MS disease classification using various algorithms.

## 6 Conclusion

To improve the accuracy of MS detection and support early clinical intervention, this study addresses critical challenges in medical imaging such as computational efficiency, generalization, and effective feature extraction through a robust hybrid classification framework. The proposed system integrates CLAHE-based contrast enhancement, deep feature learning using VGG16, and bio-inspired Whale Optimization Algorithm for optimal feature selection. This approach significantly improves the model’s ability to identify disease-related patterns in brain MRI scans. The model demonstrated exceptional performance, particularly the ANN+WOA classifier, which achieved a classification accuracy of 98%, outperforming both SVM and RF classifiers. The WOA-based feature selection effectively reduced dimensionality while retaining discriminative information, which enhanced classification accuracy and reduced computational burden even when trained on moderately sized or imbalanced datasets. This supports the system’s potential to be extended and customized for real-time clinical applications in the future. Moreover, the combination of powerful CNN-based feature extraction and intelligent optimization strikes an optimization through controlled exploration and precise exploitation, making the model both resilient and efficient. The extracted features contribute to improved decision-making and can assist healthcare professionals in diagnosing MS more accurately and at an earlier stage.

The use of WOA effectively selected the optimal feature subset, improving model performance and reducing redundancy. The proposed hybrid CNN-WOA framework not only enhances diagnostic accuracy but also confirms its potential as a robust and reliable decision-support system for MS detection. This work thus contributes a scalable, accurate, and intelligent MS classification model that can advance the field of AI-driven medical imaging.

## Data Availability

The original contributions presented in this study are included in this article/supplementary material, further inquiries can be directed to the corresponding author.
